# Emerging chemotherapy-based treatments in anaplastic thyroid cancer: an updated analysis of prospective studies

**DOI:** 10.3389/fendo.2024.1385747

**Published:** 2024-06-26

**Authors:** Bi-Cheng Wang, Guo-He Lin, Bo-Hua Kuang, Ru-Bo Cao

**Affiliations:** ^1^ Cancer Center, Union Hospital, Tongji Medical College, Huazhong University of Science and Technology, Wuhan, China; ^2^ Department of Oncology, The Second Affiliated Hospital of Anhui Medical University, Hefei, China

**Keywords:** chemotherapy, anaplastic thyroid cancer, survival outcome, response rate, adverse event

## Abstract

**Background:**

For patients with anaplastic thyroid cancer (ATC) without mutational driver genes, chemotherapy is suggested to be the first-line treatment option. However, the benefits of chemotherapy in treating ATC are limited. In this analysis, we collected the prospective data reported since 2010 to analyze the emerging chemotherapy-based treatments in ATC comprehensively.

**Methods:**

For this updated analysis, we searched PubMed (MEDLINE), Web of Science, Embase, and Cochrane CENTRAL databases from 1 January 2010 to 7 February 2024 for prospective clinical studies that contained chemotherapy-based treatments. This analysis was done to pool overall survival (OS), progression-free survival (PFS), objective response rates (ORRs), disease control rates (DCRs), and grade 3 or worse treatment-related adverse events (TRAEs).

**Results:**

Six prospective clinical trials with 232 patients were included. Chemotherapy was commonly combined with targeted therapy or radiotherapy. The pooled median OS was 6.0 months (95% CI 4.1–9.7), and the median PFS was 3.2 months (95% CI 1.9–6.0) in patients with ATC who received chemotherapy-based strategies. The integrated ORR and DCR were 21% (95% CI 15%–27%) and 64% (95% CI 55%–72%), respectively. Regarding the grade 3 or worse TRAE, the pooled incidence was 68% (95% CI 47%–86%).

**Conclusion:**

Although the emerging chemotherapy-based treatments showed antitumor activity in patients with ATC, these strategies failed to prolong the survival time substantially. More practical, safe, and novel therapeutic regimens for patients with ATC warrant further investigations.

## Introduction

Anaplastic thyroid cancer (ATC) is recognized for its rare incidence, high aggressiveness, and poor prognosis. For patients without mutational driver genes (e.g., BRAF V600E, NTRK, and RET), the recommended systemic therapeutic option in the National Comprehensive Cancer Network (NCCN) guideline was chemotherapy, including paclitaxel, doxorubicin, carboplatin, and cisplatin (1).

Retrospectively, doxorubicin plus cisplatin chemotherapy has been applied in ATC since 1985. The median overall survival (OS) was approximately 6 months, with an objective response rate (ORR) of up to 26% (2). In terms of taxane-based treatments, clinicians have begun to use these strategies since 2000. In Kenneth B. Ain’s study, the median OS was also nearly 6 months, but the ORR was over 50% (3).

Nevertheless, the efficacy of cytotoxic drugs is limited. The total lifetime of patients with ATC is hard to exceed half a year. In the “Systemic therapy: cytotoxic chemotherapy” section of the American Thyroid Association Guidelines for Management of Patients with ATC (2021), the cited latest record studying cytotoxic drugs in ATC was published in 2010 (4). We are eager to find out whether novel cytotoxic drugs have been explored to treat ATC after 2010.

Therefore, we conducted this updated analysis to collect and synthesize the efficacy and safety data of emerging chemotherapy-based treatments reported in prospective studies from January 2010 to February 2024. Through this analysis, we intend to provide more insights into future studies.

## Methods

### Literature search

For this analysis, we searched PubMed (MEDLINE), Web of Science, Embase, and Cochrane CENTRAL databases to identify prospective studies from 1 January 2010 to 7 February 2024. The search terms included “chemotherapy or taxane or paclitaxel or docetaxel or taxel or doxorubicin or cisplatin or carboplatin or mitoxantrone or cyclophosphamide or fluorouracil” and “anaplastic thyroid cancer”. The study type was defined as “clinical trial” or “randomized clinical trial”. This study was conducted according to the Preferred Reporting Items for Systematic Reviews and Meta-analyses (PRISMA) guideline (5).

### Inclusion and exclusion criteria

The inclusion criteria were as follows: (1) patients were pathologically diagnosed as ATC; (2) patients were treated with cytotoxic chemotherapy-based treatments; (3) data of survival outcomes, response rates, and incidences of treatment-related adverse events (TRAEs) were available; and (4) enrolled studies were prospective clinical trials published in English. Meeting abstracts were excluded.

### Data extraction

The following data were extracted from enrolled trials: first authors’ names, year of publication, study design, dose of drugs, median survival time, survival rates, response rates, and TRAEs. Kaplan–Meier survival curves were captured from the original studies, and the Scanlt software was used to digitize these curves.

### Statistical analysis and risk of bias assessment

Reconstructed OS and progression-free survival (PFS) curves were pooled and analyzed using the metaSurvival package of the R software. The detailed protocol has been described in Christophe Combescure’s study (6).

Pooled ORRs, disease control rates (DCRs), and incidences of TRAEs with 95% CIs were conducted using the meta package of the R software (version 4.2.2). Heterogeneity was quantified using *I* ^2^ statistic percentages (low: *I* ^2^ < 50%, *p* < 0.05). Single-arm analyses were done with the random-effects model.

Funnel plots and Egger’s test (*p* < 0.01) were used to assess publication bias.

## Results

### Basic characteristics of trials included

**Figure 1:**
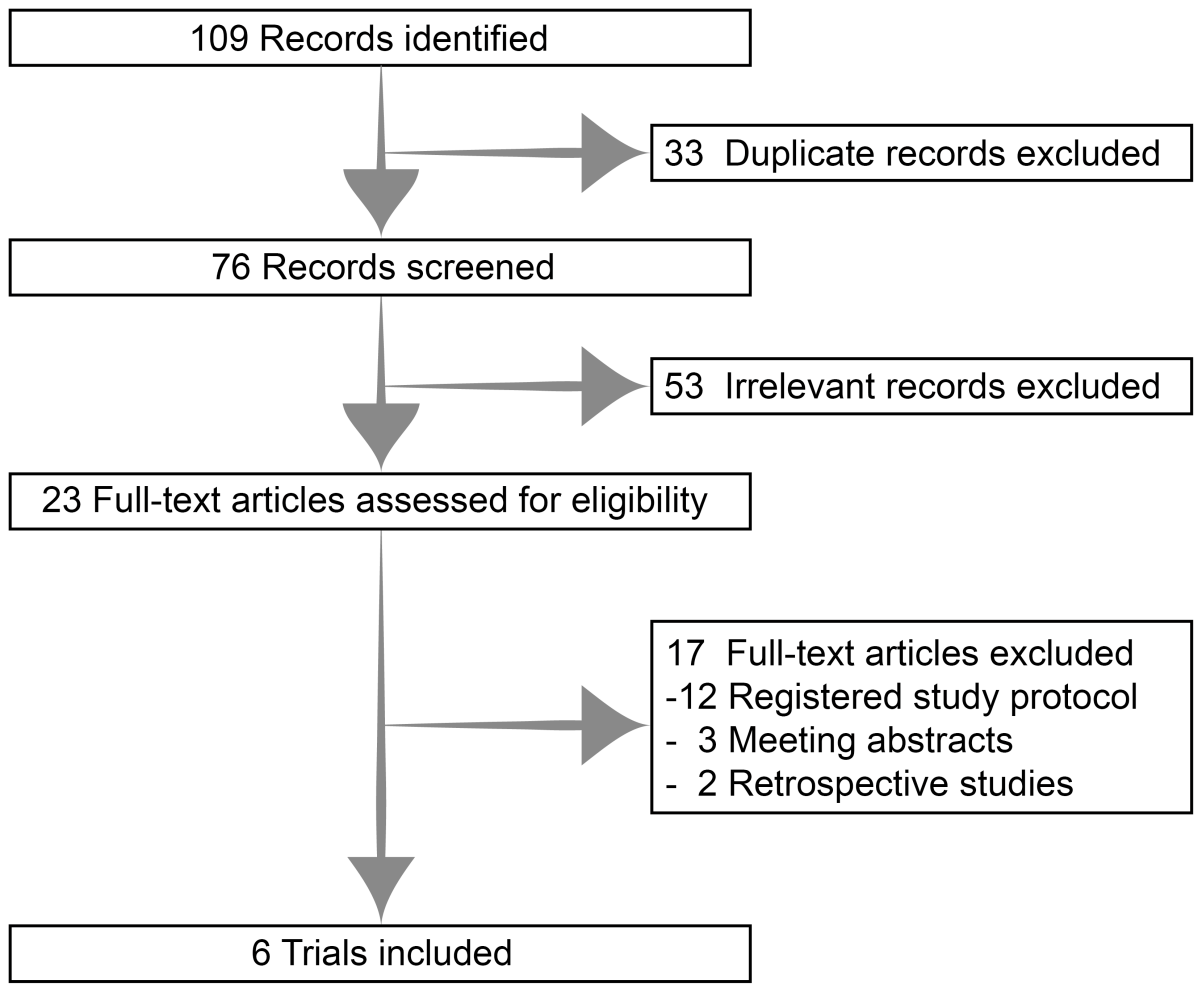
Selection process of this analysis.

The systematic search returned 109 potentially relevant records. After deduplication and screening, six clinical trials comprising 232 patients were identified and included in the analysis (**Figure 1**) (7–12).

**Table 1** summarizes the characteristics of the trials. Median age ranged from 56 to 71. Cytotoxic drugs included paclitaxel, docetaxel, doxorubicin, and carboplatin. The combination of chemotherapy with targeted therapy (pazopanib, fosbretabulin, and efatutazone) was applied in three trials. Although radiotherapy was pre-designed in three of the six trials (7–9), patients in the other three trials received radiotherapy either (10–12).

**Table 1:** Characteristics of enrolled studies of chemotherapy in anaplastic thyroid cancer.

Study	Publication year	Design	Group	No. of patients	Median age (range)	Drugs
Eric J. Sherman	2023	A double-blind, multicenter, randomized, phase 2 prospective trial	Paclitaxel plus pazopanib and radiotherapy Paclitaxel plus placebo and radiotherapy	36 35	65 63	Paclitaxel: 80 mg/m^2^ weekly (50 mg/m^2^ weekly concurrent) Pazopanib: 400 mg orally daily Radiotherapy: 66 Gy/33 Fractions
Ashish V. Chintakuntlawar	2019	An open-label, two-cohort, phase 2 prospective study	Docetaxel + doxorubicin plus pembrolizumab and radiotherapy	3	56	Docetaxel: 20 mg/m^2^ weekly Doxorubicin: 20 mg/m^2^ weekly Pembrolizumab: 200 mg every 3 weeks Radiotherapy: 66 Gy/33 Fractions
Naoyoshi Onoda	2016	An open-label, multicenter, prospective study	Paclitaxel plus radiotherapy	56	71 (47–84)	Paclitaxel: 80 mg/m^2^ weekly Radiotherapy: 40 Gy or 60 Gy
Julie A. Sosa	2014	An open-label, multicenter, randomized controlled, prospective study	Paclitaxel + carboplatin plus fosbretabulin Paclitaxel + carboplatin	55 25	63 (28–83) 62 (33–82)	Paclitaxel: 200 mg/m^2^ every 3 weeks Carboplatin: AUC 6 every 3 weeks Fosbretabulin: 60 mg/m^2^ d1, 8, and 15 every 3 weeks
R. C. Smallridge	2013	An open-label, multicenter, phase 1 prospective study	Paclitaxel plus efatutazone	15	59 (43–82)	Paclitaxel: 175 mg/m^2^ every 3 weeks Efatutazone: 0.15/0.3/0.5 mg orally twice daily
Kenji Kawada	2010	An open-label, single-center, prospective study	Docetaxel	7	68 (66–78)	Docetaxel: 60 mg/m^2^ every 3 weeks

Survival outcomes reported in the trials are summarized in **Table 2**. The median OS ranged from 2.8 months (docetaxel + doxorubicin plus pembrolizumab and radiotherapy) to 7.3 months (paclitaxel plus placebo and radiotherapy), the median PFS ranged from 1.4 months (docetaxel) to 3.3 months (paclitaxel + carboplatin plus fosbretabulin), and the 1-year survival rates ranged from 8.7% (paclitaxel + carboplatin) to 37.1% (paclitaxel plus pazopanib and radiotherapy).

**Table 2:** Survival outcomes of anaplastic thyroid cancer patients treated with chemotherapy-based treatments.

Study	OS (95% CI)	PFS (95% CI)	1 year survival rate
Eric J. Sherman 2023	Experimental group: 5.7 months (4.0–12.8) Control group: 7.3 months (4.3–10.6)	NR	37.1% 29.0%
Ashish V. Chintakuntlawar 2019	2.8 months	NR	NR
Naoyoshi Onoda 2016	6.7 months (4.4–9.0)	1.6 months	26.8%
Julie A. Sosa 2014	Experimental group: 5.2 months (3.1–9.0) Control group: 4.0 months (2.8–6.2)	3.3 months (2.3–5.6) 3.1 months (2.7–5.4)	25.9% 8.7%
R. C. Smallridge 2013	NR	NR	NR
Kenji Kawada 2010	3.0 months (1.6–24.3)	1.4 months (0.2–11.7)	14.3%

OS, overall survival; PFS, progression-free survival; CI, confidence interval; NR, not reported.

### Reconstructed survival outcomes

**Figure 2:**
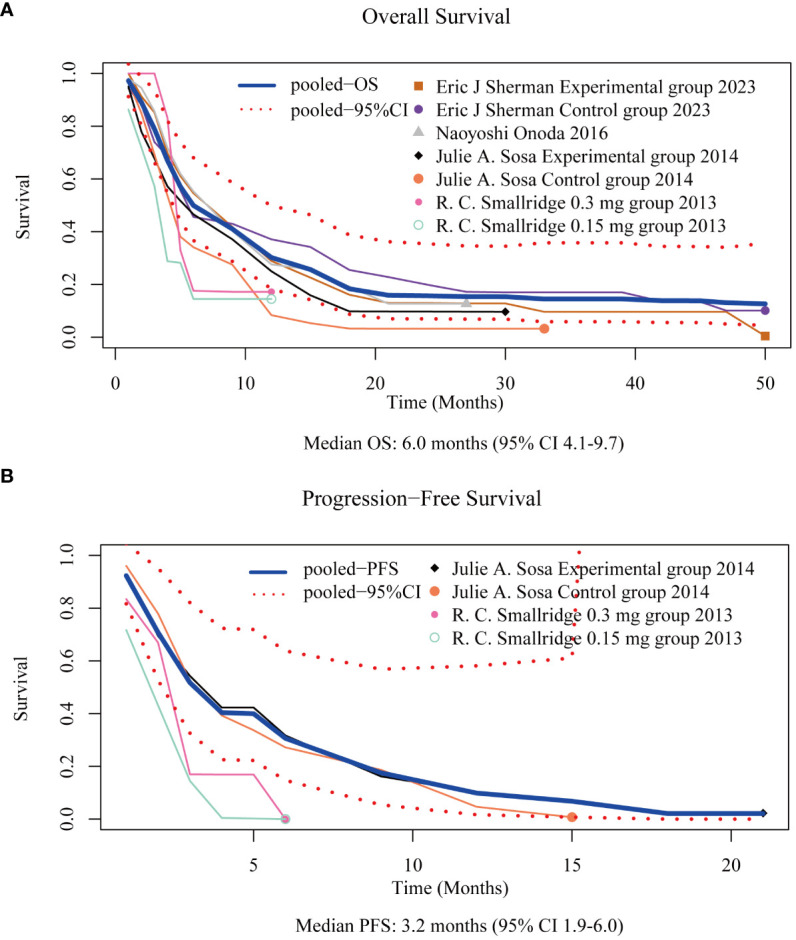
Reconstructed overall survival [OS, **(A)**] and progression-free survival [PFS, **(B)**] curves.

OS data were derived from the OS curves reported in four of the six trials (7, 9–11). The pooled median OS was 6.0 months (95% CI 4.1–9.7) (**Figure 2A**). The 6-month and 1-year OS rates were 50% (95% CI 37–68) and 30% (95% CI 18–50), respectively.

PFS data were reconstructed from the PFS curves published in two of the six trials (10, 11). The pooled median PFS was 3.2 months (95% CI 1.9–6.0) (**Figure 2B**). The 6-month and 1-year PFS rates were 31% (95% CI 10–64) and 10% (95% CI 1–58), respectively.

### Responses and toxicities

**Figure 3:**
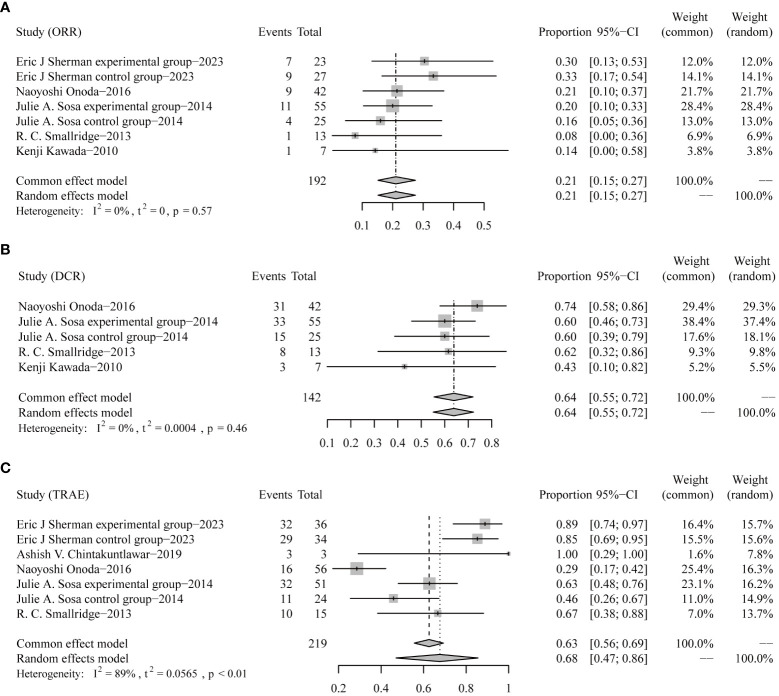
Forest plots for objective response rates [ORRs, **(A)**], disease control rates [DCRs, **(B)**], and incidences of grade 3 or worse treatment-related adverse events [TRAEs, **(C)**].

Similarly, response rates and grade 3 or worse safety data were pooled-analyzed. A total of 192 patients from five trials were enrolled in the analysis of ORR (7, 9–12). The pooled ORR was 21% (95% CI 15–27) (**Figure 3A**). DCR data from 142 patients in four trials were integrated (9–12). The pooled DCR was 64% (95% CI 55–72) (**Figure 3B**).

In terms of TRAE data collected from five trials involving 219 patients (7–11), the pooled rate of grade 3 or worse TRAE was 68% (95% CI 47–86) (**Figure 3C**).

### Risk of bias

**Figure 4:**
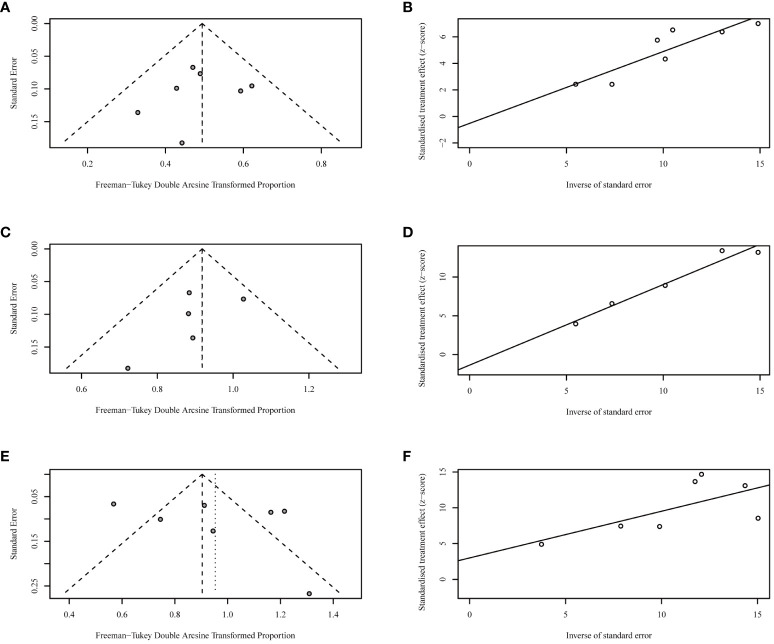
Funnel plots and Egger’s tests for objective response rates [ORRs, **(A, B)**], disease control rates [DCRs, **(C, D)**], and incidences of grade 3 or worse treatment-related adverse events [TRAEs, **(E, F)**].

Funnel plots and Egger’s tests failed to find any publication bias during the analyses of ORR (**Figures 4A, B**), DCR (**Figures 4C, D**), and TRAE (**Figures 4E, F**).

## Discussion

In this updated analysis of emerging chemotherapy-based treatment for patients with ATC, the total lifetime remains within 6 months even if targeted therapy, immunotherapy, or radiotherapy were added, and DCR was over 60%. To overcome the issues, basic researchers and physicians still need to do numerous explorations.

Among the eligible trials, cytotoxic drugs included only paclitaxel, docetaxel, doxorubicin, and carboplatin. Such selections indicate that clinicians or the drug industry may be more confident in these drugs. According to the search strategy described in the Methods section, mitoxantrone, cyclophosphamide, and fluorouracil have also been administered in patients with ATC (13–15). However, substantial improvements in survival outcomes were not identified when patients with ATC were treated with these drugs. Since novel-designed cytotoxic drugs have been approved to treat both solid and hematological tumors, like antibody–drug conjugates brentuximab vedotin (16) and sacituzumab govitecan (17), whether antibody–drug conjugates can help to enhance the responses and prolong survival outcome in ATC deserves further investigations.

In the trials enrolled in this analysis, we found that nearly all participants have received radiotherapy. Indirectly, radiotherapy is critically essential for patients with ATC to achieve local disease control since a rapidly growing neck mass can cause asphyxiation. In other solid tumors, radiotherapy has been certified to benefit patients with a tolerable safety profile. In patients with renal cell carcinoma who decline surgery, stereotactic body radiotherapy can be suggested as a safe and effective standard treatment option (18). In advanced lung cancer, the addition of radiotherapy significantly improved response rates compared with immunotherapy alone (19). Therefore, we believe that reasonable palliative radiotherapy may bring unexpected benefits for patients with advanced ATC.

Although immunotherapy has significantly impacted previous therapeutic strategies for patients with cancer, the efficacy of immune checkpoint inhibitors on ATC was limited. In the subset of patients with PD-L1 < 1%, the median OS was 1.6 months (95% CI 1.0–19.6), while for the subset of patients with PD-L1 > 1%, the data were not reached (32364844). Clinicians have started investigating the combination of immunotherapy and radiotherapy (20, 21). In head and neck cancer, a synergistic effect has been found as both irradiated sites and metastatic lesions achieved responses (21). Accordingly, digging for effective combination therapies for patients with ATC is also crucial.

Regarding the grade 3 or worse TRAEs, the incidence (68%) deserves our attention. Increased alanine aminotransferase, dermatitis radiation, dysphagia, and neutropenia were the most common grade 3 or worse TRAEs recorded in the enrolled trials.

## Conclusion

In this updated analysis, the emerging chemotherapy-based treatments did not substantially prolong the survival time of patients with ATC. Novel cytotoxic or targeted drugs or creative combination therapies are warranted in future studies.

## Author contributions

B-CW: Conceptualization, Data curation, Formal Analysis, Funding acquisition, Investigation, Methodology, Project administration, Resources, Software, Supervision, Validation, Visualization, Writing – original draft, Writing – review & editing. G-HL: Data curation, Supervision, Validation, Visualization, Writing – original draft, Writing – review & editing. B-HK: Formal Analysis, Investigation, Methodology, Software, Writing – original draft, Writing – review & editing. R-BC: Formal Analysis, Investigation, Methodology, Project administration, Software, Writing – original draft, Writing – review & editing.
